# Alteration of the Functional Connectivity of the Cortical Areas Characterized by the Presence of Von Economo Neurons in Schizophrenia, a Pilot Study

**DOI:** 10.3390/jcm12041377

**Published:** 2023-02-09

**Authors:** Claudio Brasso, Mario Stanziano, Francesca Marina Bosco, Rosalba Morese, Maria Consuelo Valentini, Alessandro Vercelli, Paola Rocca

**Affiliations:** 1 Department of Neuroscience “Rita Levi Montalcini”, University of Turin, 10126 Turin, Italy; 2 Struttura Complessa di Psichiatria Universitaria, Dipartimento di Neuroscienze e Salute Mentale, Azienda Ospedaliero-Universitaria “Città della Salute e della Scienza di Torino”, 10126 Turin, Italy; 3 Neuroradiology Unit, Fondazione IRCCS Istituto Neurologico “Carlo Besta”, 20133 Milan, Italy; 4 Research Group on Inferential Processes in Social Interaction (GIPSI), Department of Psychology, University of Turin, 10124 Turin, Italy; 5 Faculty of Communication Sciences, Università della Svizzera Italiana, 6900 Lugano, Switzerland; 6 Faculty of Biomedical Sciences, Università della Svizzera Italiana, 6900 Lugano, Switzerland; 7 Struttura Complessa di Neuroradiologia, Dipartimento Diagnostica per Immagini e Radiologia Interventistica, Azienda Ospedaliero-Universitaria “Città della Salute e della Scienza di Torino”, 10126 Turin, Italy; 8 Neuroscience Institute Cavalieri Ottolenghi (NICO), 10043 Orbassano, Italy

**Keywords:** von Economo neurons, VENs, fMRI, schizophrenia, resting-state functional connectivity, salience network, negative symptoms, functioning, anterior insula, ventral tegmental area

## Abstract

Von Economo neurons (VENs) are rod, stick, or corkscrew cells mostly located in layer V of the frontoinsular and anterior cingulate cortices. VENs are projection neurons related to human-like social cognitive abilities. Post-mortem histological studies found VEN alterations in several neuropsychiatric disorders, including schizophrenia (SZ). This pilot study aimed to evaluate the role of VEN-containing areas in shaping patterns of resting-state brain activation in patients with SZ (n = 20) compared to healthy controls (HCs; n = 20). We performed a functional connectivity analysis seeded in the cortical areas with the highest density of VENs followed by fuzzy clustering. The alterations found in the SZ group were correlated with psychopathological, cognitive, and functioning variables. We found a frontotemporal network that was shared by four clusters overlapping with the salience, superior-frontal, orbitofrontal, and central executive networks. Differences between the HC and SZ groups emerged only in the salience network. The functional connectivity of the right anterior insula and ventral tegmental area within this network were negatively correlated with experiential negative symptoms and positively correlated with functioning. This study provides some evidence to show that in vivo, VEN-enriched cortical areas are associated with an altered resting-state brain activity in people with SZ.

## 1. Introduction

Von Economo neurons (VENs) were described by von Economo and Koskinas [1] as layer V large rod cells, stick cells, or corkscrew cells, mostly located in the frontoinsular (FI) and anterior cingulate cortex (ACC) [2,3,4,5]. VENs have a large fusiform cell body, which is larger than that of the pyramidal neurons in the same layer. Their dendritic processes extend perpendicularly from the apical and basal poles of the cell body to the pial surface and are single, broad, and low-branched [6,7,8]. The soma has a cylindrical appearance and the basal part of the body axis is usually helical [6,8,9,10]. They are a regionally distinctive type of extra-telencephalic neuron that shows specific intrinsic membrane properties and represents 5% of projection neurons in layer V [11]. VENs are excitatory neurons that are positive for Microtubule-Associated Protein 2 (MAP2), monoclonal antibody, and neurofilament protein (SMI-32) [3,9] and can be stained with DiI inserted into the cortical white matter. These characteristics, which have been confirmed at a transcriptomic level [11,12], indicate the long-range connectivity of this kind of neuron [9,10,13] that projects to subcerebral targets [11]. VENs are present in ancestral mammals in the context of specific adaptive pressures and evolved only in a restricted number of species [14]. In particular, they have been identified in several cetaceans, elephants, and some primates, namely chimpanzees, bonobos, gorillas, orangutans, macaques, and humans [15]. Their distribution in great apes seems to correlate with human-like social cognitive abilities and self-awareness [15]. This phylogenetic distribution may suggest a correlation between VENs, brain size, and the “social brain” [15]. In homo sapiens, VENs appear in prenatal development (week 35) and increase until 4 years of age [10,16].

A recent study that fully described the transcriptome differences between pyramidal neurons and VENs in the ACC (layer V) showed that out of the top 20 genes with higher expression of VENs, about half of them had known associations with human social-emotional disorders such as autistic spectrum disorder (ASD), schizophrenia (SZ), Alzheimer’s disease (AD), and major depressive disorder [17].

Many studies have analyzed VENs in post-mortem brain samples by comparing subjects diagnosed with neuropsychiatric disorders and healthy controls (HCs). Differences were found in VENs’ density, structural, and ultrastructural characteristics in sporadic amyotrophic lateral sclerosis [18], AD [19], behavioral variant frontotemporal dementia (bv-FTD) [20,21], ASD [22,23], alcohol use disorder [24], and SZ [25,26,27]. Focusing on the associations between VENs and SZ in histological post-mortem studies, it has been demonstrated that the density of VENs in the right ACC (R-ACC) correlated positively with the age at onset and inversely with the duration of the illness (DOI) [25]. As patients with early-onset SZ and longer DOI showed reduced VEN density, it could be speculated that these particular neurons play a role in neurodevelopmental and neurodegenerative processes linked to SZ pathophysiology [25]. At an ultrastructural level, a higher concentration of lysosomal aggregates was found in VENs in layer Vb in the ACC of subjects with SZ compared to those found in the pyramidal cells of the same patients and VENs in the same cerebral area in HCs. In addition, in this case, a neurodegenerative process affecting selective VENs was linked to SZ [27]. On the contrary, VENs are overabundant in super-agers, i.e., elderly subjects usually aged 80 or over, who present cognitive abilities far above what is age-expected [28]. In this case, an overabundance of VENs may represent a resilience factor to the conventional pathways of brain aging.

Based on the evidence of the alteration of VENs in patients with SZ in post-mortem studies, we decided to investigate the putative role of these neurons in vivo using magnetic resonance (MR) imaging. To achieve this aim, we followed the methodology proposed by Cauda and colleagues [29] by performing a functional connectivity analysis based on regions of interest (ROIs) that comprise the cortical areas with the highest density of VENs. In the present study, this analysis was performed on a clinical sample of patients with SZ compared with a group of HCs. In addition, we assessed the presence of any significant relationships between the potential differences in functional connectivity found in the group of patients with psychopathological, cognitive, and functioning variables. We expected to find significant differences in the resting-state functional connectivity (rsFC) between the two groups and that some of these differences would correlate with the symptoms, cognitive deficits, and level of functioning.

## 2. Materials and Methods

### 2.1. Participants

Twenty individuals with a diagnosis of SZ according to DSM-5 criteria [30] and twenty HCs matched for age, sex, and education were included in the study. All participants took part in the study voluntarily after giving their informed consent. The study was approved by the Local Research Ethics Committee (protocol number: 0076364). All participants met the following inclusion criteria: (1) aged between 18 and 65 years old; (2) right-handed; (3) no history of neurological illness; and (4) demonstrate basic cognitive and linguistic abilities by achieving a score > 70 in the Test di Intelligenza Breve (TIB; cutoff score 70) [31], the Italian equivalent of the National Adult Reading Test (NART) [32]. Patients with SZ met the following criteria: (1) no other concomitant mental disorder, and (2) clinically stable, i.e., no hospitalization and antipsychotic treatment modification in terms of drug and dosage in the last three months. HCs met the following criteria: (1) no current use of psychoactive drugs, and (2) no personal and familial history of mental disorders.

### 2.2. Clinical and Cognitive Assessment

Symptom severity was rated with the Positive and Negative Syndrome Scale (PANSS) [33]. We adopted the solution proposed by Wallwork et al. [34] to calculate the scores for the dimensions “disorganization” and “positive symptoms”. The Italian version of the Brief Negative Symptoms Scale (BNSS) was employed to assess negative symptoms [35]. These symptoms were grouped into two factors: “avolition”, consisting of anhedonia, asociality, and avolition, and “expressive deficit”, including blunted affect and alogia [36,37]. Depressive symptoms were rated with the Calgary Depression Scale for Schizophrenia (CDSS) [38]. The Personal and Social Performance Scale (PSP) was used to evaluate the level of functioning [39]. Antipsychotic dosage was converted to the chlorpromazine (CPZ)-equivalent dose according to the equivalences proposed by Leucht et al. [40]. Extrapyramidal symptoms were rated with the Simpson–Angus Scale (SAS) [41].

The principal neurocognitive domains were assessed with the Brief Assessment of Cognition in Schizophrenia (BACS) [42]. The Italian version of the Strange Stories Test (SST) was employed to evaluate the theory of mind (ToM) abilities [43].

### 2.3. fMRI data Processing and Analysis

#### 2.3.1. Image Acquisition

MRI data were collected in a single session for each subject using a 3.0 T MRI Scanner (Philips Ingenia, Philips, Amsterdam, Netherlands). We employed a 32-channel array head coil (Resonance Tecnology, Inc., Northridge, Los Angeles, CA, USA). MRI data were acquired at the Neuroimaging Center (Centro di Neuroimmagini–CNI) of the Neuroscience Institute of Turin (NIT) at the University of Turin, located in the Azienda Ospedaliera Universitaria “Città della Salute e della Scienza di Torino” in Turin, Italy. Head movements were restricted using foam cushions. Resting-state functional images were acquired using a T2*-weighted GRE-EPI sequence (TR = 2000 ms; TE = 40 ms; thickness = 4 mm; matrix size = 128 × 128; FOV = 230 mm; voxel = 3.6 mm^2^), providing 32 interleaved images per volume, parallel to the AC–PC line and covering the whole brain. Each fMRI series consisted of 368 images, the first 4 of which were discarded to allow the scanner to reach a steady state. Structural images were recorded applying a T1-weighted sequence with the following characteristics: TR = 8.1 ms, TI = 900 ms, TE = 3.7 ms, voxel size = 1 × 1 × 1 mm^3^. Structural images were acquired for functional image registration and normalization. Scanner noise was continuous throughout the experiment, providing a constant auditory background, and attenuated with ear defenders and headphones.

Subjects were asked to keep their eyes closed during scanning. They were also asked to think about nothing in particular and not to fall asleep. After the scanning session, participants were asked if they had fallen asleep in the scanner. Subjects who replied positively or were not sure about the answer to this question were excluded from the study. The habitual pharmacological treatment of patients was not discontinued at the time of the MRI scanning session. Smokers were allowed to smoke before MRI scanning (last cigarette approximately 60 min before the session) to avoid the potential effects of nicotine withdrawal.

#### 2.3.2. fMRI Data Pre-Processing

Image data were pre-processed with the BrainVoyager QX 2.6.3 software (Brain Innovation, Maastricht, The Netherlands). Preprocessing of fMRI data included the following corrections: slice-scan timing acquisition, 3D rigid body motion, linear trend removal, and temporal high-pass filtering (cutoff = 3 cycles per time course).

As head motion can affect fMRI data, we conservatively excluded subjects whose time series exceeded 2 mm in translation and/or 2 degrees in rotation [44]. Furthermore, to assess the potential differences between patients’ and healthy controls’ micro-movements, we applied the algorithm proposed by Power et al., [45]. Functional imaging data of each subject were automatically coregistered to their corresponding structural data, spatially normalized to the standard Talairach space. This normalization was performed using a 12-parameter affine transformation, smoothed with an 8 mm full-width-half-maximum (FWHM) isotropic Gaussian kernel and resampled to an isometric 3 mm grid covering the entire Talairach box.

To improve the specificity of the results, several spurious or nonspecific sources of blood-oxygen-level-dependent (BOLD) signal variance were regressed out from each time series as they were unlikely to be relevant to neuronal activity [45]. These nuisance factors included the six head-motion parameters (translation along and rotation around the three orthogonal axes) estimated during the initial rigid-body registration procedure, as well as the average signal time course from ventricles (CSF) and white matter (WM). These average signals were determined according to anatomical boundaries from each participant’s 3D T1 structural images [46]. As suggested by Chai et al. [47], to minimize the partial volume effects with grey matter (GM), the WM and CSF masks were reduced by one voxel, thus resulting in substantially smaller masks than in the original segmentations [47]. We did not regress the signal from the GM voxels (part of the so-called “global signal”) to reduce the risk of artificially introducing or overestimating spurious anticorrelations [48].

Finally, we applied a temporal band-pass filter of 0.008–0.09 Hz on the time series to restrict the analysis to low-frequency fluctuations, which characterize fMRI BOLD resting-state activity [49].

#### 2.3.3. Definition of the Regions of Interest

We based the choice of the regions of interest (ROIs) on two principles: (1) following the methodology of the most specific study on the brain resting-state functional connectivity based on the areas with the highest densities of VENs, i.e., Cauda et al. (2013) [29], and (2) delimiting as precisely as possible the grey matter areas with the highest concentration of VENs. Contrary to Cauda et al. (2013), which employed a 1.5 T scanner, we had at our disposal a static magnetic field of 3T. This difference allowed us to obtain higher-resolution images. Therefore, we were able to define smaller and more precise ROIs, including the cortical areas with the highest densities of VENs. The coordinates proposed by Cauda et al. [29] were included within the bilateral ventral agranular insulae and bilateral rostroventral areas 24, as described in the Human Brainnetome Atlas [50]. Therefore, two expert neuroradiologists (M.C.V./M.S.) followed the cortical parceling proposed in the Human Brainnetome Atlas [50] to select the gray matter of the bilateral ventral agranular insulae and rostroventral areas 24. Then, following the anatomic and histological atlas by Allmann et al. [16], they delimited in both hemispheres the fronto-insular cortex (FIC) within the agranular insula and limbic anterior cingulate cortex (l-ACC) within the rostroventral area 24. The FIC comprises the anterior short and accessory insular gyri [51] and the l-ACC corresponds to the Brodmann area 24b [3,52]. The Brodmann area 24b is located on the surface of the anterior cingulate gyrus between the callosal sulcus and the cingulate sulcus, rostral to the genu of the corpus callosum. It is bounded rostrally by area 24c, caudally by area 33o, and ventrally by area 24a [3,52]. M.C.V. and M.S. selected the ROIs of each subject together in order to agree on the boundaries of each selected area.

**Figure 1:**
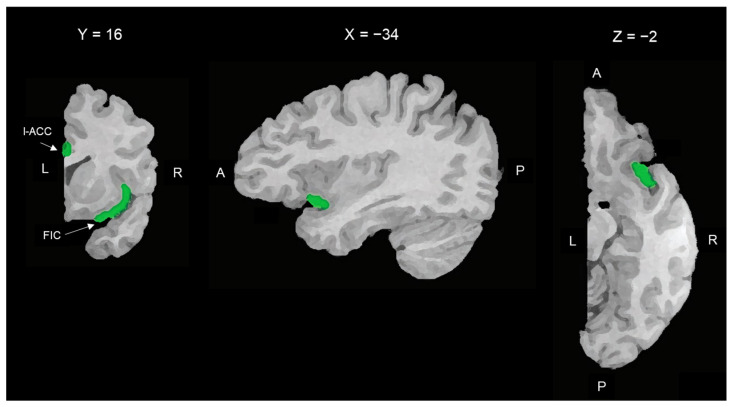
Spatial location of the right ROIs used as the seed region for rsFC analysis. From left to right: coronal, parasagittal, and transversal slices of the right hemisphere. ROIs are indicated in green. l-ACC: limbic anterior cortical cortex (Brodmann area 24b); FIC: fronto-insular cortex (anterior short and accessory gyri of the anterior insula). L = left; R = right; A = anterior; P = posterior.

The two ROIs of the right hemisphere are shown in Figure 1.

#### 2.3.4. Functional Connectivity Analysis

The bilateral FIC and l-ACC were used as the seed region for the whole-brain functional connectivity analysis. The seed-based approach extracts the averaged time series from the voxels of the seed region and measures the temporal correlation between this signal and the time series from all other brain voxels. An rsFC map was computed for each subject using the seed time course as a regressor. Fits to the model were evaluated after removing the auto-regression factor. The resulting single-subject t-maps were then submitted to a second-level random-effects analysis to create a random-effects whole-sample map (SZ patients and HCs pooled together). An analysis of covariance (ANCOVA) was carried out controlling for age, gender, duration of illness, and CPZ-equivalent effects [29].

As proposed by a previous study on rsFC of cortical areas characterized by von Economo neurons [29], a voxel-wise unsupervised fuzzy clustering technique was employed on the ROI-generated functional networks (VEN networks) to evaluate whether these networks could be divided into two or more subclusters [53]. Fuzzy clustering groups a set of voxels into clusters of activation [53]. The voxels were grouped into clusters sharing a similar temporal profile, which suggests a functional similarity. The z-standardized signal time courses of all voxels were considered simultaneously, compared, and assigned to representative cluster time courses (cluster centroids). The extent to which a voxel belonged to a cluster was defined by the similarity (measured by correlation) of its time course to the cluster centroid. This process identified clusters containing a certain number of voxels that shared a similar temporal profile (standardized time course). In this method, “fuzziness” refers to the fact that single voxels are usually not uniquely assigned to one single cluster but can instead be classified in more than one cluster. Following the work of Cauda et al. [29], the number of clusters was set to four and the degree of fuzziness was controlled by setting the “m” parameter to 0.4, thus allowing some voxels to be classified in more than one cluster. Dataset dimensionality was reduced using a principal component analysis that captured at least 90% of the total variance/covariance. Using the SogIca method [29], the single-subject maps were pooled together and the probabilistic maps, reported in the interval [10–100%], were employed to represent these group-level results on the inflated representation of a template brain (average brain). This phase of the analysis was carried out on the whole sample (HCs and SZ). The four resulting probabilistic maps were used as the search volume for the between-group (HC vs. SZ) comparison.

To correct for multiple comparisons, regional effects were considered significant only for clusters exceeding a minimum size determined with a non-parametric randomization approach (cluster-level correction). Starting with an initial voxel-level threshold of *p* = 0.05 (uncorrected), a minimum cluster size was estimated after 1.000 Monte Carlo simulations, which protected against false-positive clusters up to 5% [54]. This led to a cluster threshold K > 22 voxels in the native resolution. Statistical analysis of the significant main effects was performed within BrainVoyager.

Within the group of patients with SZ, individual averaged values of the BOLD signal magnitude of significant clusters were employed in correlation analyses with clinical scores [55]. This analysis was performed with the Statistical Package for Social Science (SPSS) Version 27.0.

### 2.4. Clinical Association with Neuroimaging and Between-Group Comparison of Sociodemographic and Cognitive Variables

Within the group of patients with SZ, individual averaged values of the BOLD signal magnitude of significant clusters were correlated (Pearson correlations) with individual ratings of symptoms, cognitive performance, and psychosocial functioning [55]. Bonferroni’s correction was applied for multiple comparisons. Between-group comparison of sociodemographic, neurocognitive, and ToM variables was performed with a one-way ANOVA. These analyses were performed with the Statistical Package for the Social Sciences, SPSS, version 27.0 for Windows (SPSS, Chicago, IL, USA), with statistical significance set at *p* = 0.05.

## 3. Results

### 3.1. Demographic, Clinical, and Cognitive Characteristics of the Sample

The demographic characteristics of the SZ and HC groups were homogeneous (Table 1).

The mean age in the SZ group was 41.5 years and 42.1 in the HC group. In both groups, 13 subjects were males and 7 were females. The SZ and HC groups received, on average, 13.7 and 13.9 years of schooling. We observed no statistical differences in the between-group comparison of the sociodemographic variables.

The clinical characteristics of the SZ group are summarized in Table 1. The age at onset and the duration of illness were heterogeneous with a mean of 27.5 and 14.4 years, respectively. The scores of the positive and disorganization dimensions were globally low, with averages of 7.45 and 6.25, respectively. Higher scores were observed in the avolition dimension (mean score 21.85) and, to a lesser extent, in the expressive deficit factor (mean score 11.65). Depressive symptoms were globally low (mean score 4.1 on the CDSS). Personal and social functioning was partly impaired with a mean PSP score of 61.6. The CPZ-equivalent daily dose was 371.8 mg, with a standard deviation of 144.87 mg. Eighteen subjects with SZ were in treatment with an atypical antipsychotic drug, whereas two patients were taking typical antipsychotic drugs. None of them was using clozapine. All patients were treated with only one antipsychotic drug at a dosage recommended in the datasheet for the treatment of SZ. The extrapyramidal side effects were negligible (mean SAS = 0.75). Regarding the concomitant psychopharmacological treatments, six patients were treated with a low or medium dose of an antidepressant (five with a selective serotonin reuptake inhibitor and one with the serotonin modulator vortioxetine), four with a benzodiazepine, and one with the nonbenzodiazepine zolpidem. Regarding antidepressants, two patients were treated with escitalopram 10 mg/d, one with paroxetine 10 mg/d, one with sertraline 50 mg/d, one with fluvoxamine 50 mg/d, and one with vortioxetine 5 mg/d. All patients took the antidepressant after breakfast. Focusing on benzodiazepines and zolpidem, one patient was treated with estazolam 1 mg, one with flurazepam 30 mg, two with alprazolam 1 mg, and one with zolpidem 10 mg. All patients took these drugs for the treatment of insomnia. The fMRI scan was performed between 3 and 5 p.m., which was 15–17 h after the last dose. Patients were not treated with other concomitant psychoactive medications.

Neurocognitive performance, evaluated using the BACS, and ToM abilities, measured using the SST, are shown in Table 1. Compared to HCs, SZ patients showed cognitive deficits in all cognitive domains explored, except for the executive functions assessed using the Tower of London test.

### 3.2. RsFC and Fuzzy Clustering Results

**Figure 2:**
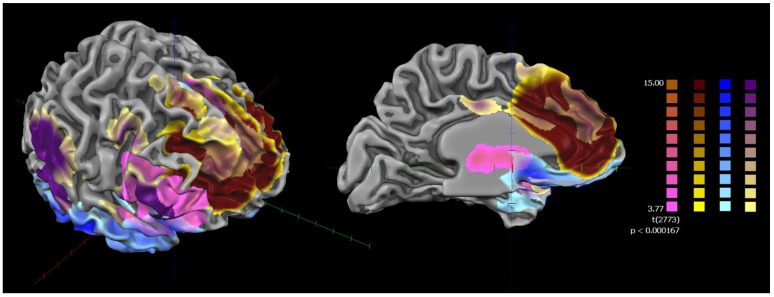
ROI-based rsFC of cortical areas with the highest densities of VENs used as the seed (whole sample: HCs + patients with SZ). HCs: healthy controls; SZ: patients with schizophrenia; ROI-based rsFC of cortical areas with the highest densities of VENs used as the seed. Random-effects whole-sample map resulting from analysis of covariance (ANCOVA), controlling for age, gender, duration of illness, and chlorpromazine equivalents. Corrections for multiple comparisons performed at the cluster level using 1.000 Monte Carlo simulations (*p* < 0.05), leading to a cluster threshold of K > 22 voxels in the native resolution. The different colors represent the subclusters derived from the voxel-wise parceling of the ROI-generated functional network (fuzzy clustering).

None of the subjects were excluded from falling asleep during the scan. The results of the rsFC of the bilateral FIC and bilateral l-ACC in the whole sample (HCs and SZ) showed that they were part of a frontotemporal network mainly involving the bilateral ACC, insulae, and prefrontal and supplementary motor cortices (Figure 2).

**Figure 3:**
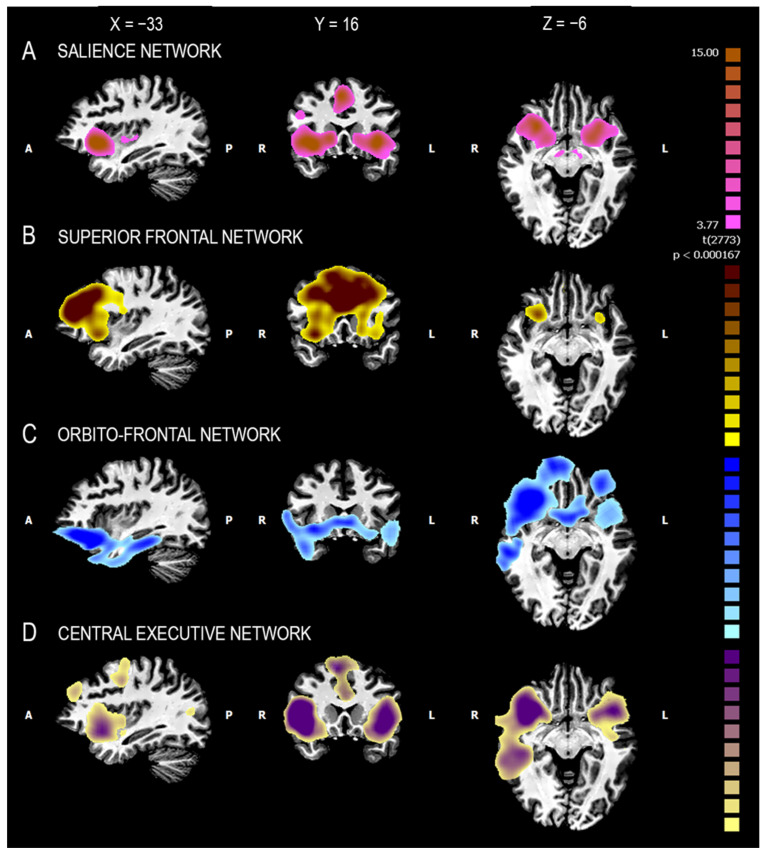
Two-dimensional maps of the subnetworks resulting from the voxel-wise parceling of the ROI-generated functional network (fuzzy clustering) of the whole sample (HCs + SZ). HCs: healthy controls; SZ: patients with schizophrenia. Connectivity-based parceling of the VEN-derived functional network. Each panel represents a probabilistic map of one of the four functional connectivity-defined subclusters. These subclusters were associated with four well-known functional networks: the salience network (**A**), the superior frontal network (**B**), the orbitofrontal network (**C**), and the central executive network (**D**). L = left; R = right; A = anterior; P = posterior.

Voxel-wise parceling (fuzzy clustering) decomposed the functional network into four clusters. These clusters were associated with four well-known functional networks, namely the salience network (ACC and insula), the superior frontal network (dorsomedial frontal cortex), the orbitofrontal network (ventromedial prefrontal cortex), and the central executive network (ACC, insula, dorsolateral prefrontal cortex, and supplementary motor area) (Figure 2 and Figure 3) [56].

**Figure 4:**
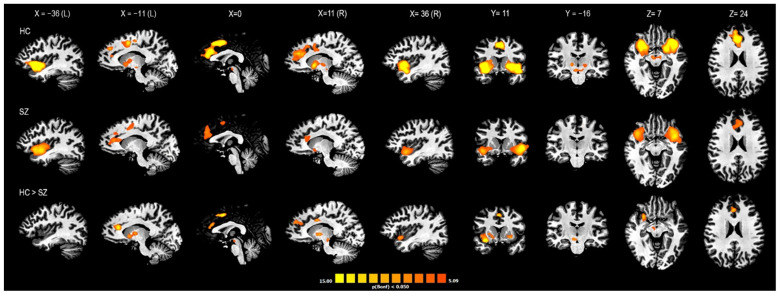
**Between-group comparison of rsFC within the salience subnetwork.** Two-dimensional representation of the subcluster belonging to the salience network derived from the fuzzy clustering analysis of the VEN-derived functional connectivity network. HC: maps of healthy controls; SZ: maps of patients with schizophrenia; HC > SZ: maps of the HC versus SZ between-group contrast. The areas shown in the HC > SZ maps indicate higher connectivity of the salience network in terms of the extension and intensity of the functional cluster in the HC group compared to the SZ group. These areas correspond to the right anterior insula, the bilateral dorsal ACC/pre-supplementary motor areas, the right ventral striatum, and the right ventral tegmental area (R-VTA). Corrections for multiple comparisons performed at the cluster level using 1.000 Monte Carlo simulations (*p* < 0.05), leading to a cluster threshold of K > 22 voxels in the native resolution.

From the between-group (HC vs. SZ) comparison, statistically significant differences were found within the salience network (SN) cluster only. The SZ group showed lower connectivity of the SN in terms of the extension and intensity of the functional cluster. In particular, compared to HCs, patients with SZ showed lower connectivity values in four subclusters within the SN cluster corresponding to the right anterior insula (R-AI), the bilateral dorsal ACC (d-ACC)/pre-supplementary motor areas (pre-SMA), the right ventral striatum (R-vSr), and the right ventral tegmental area (R-VTA; Figure 4).

The correlations between the individual rsFC values of the four above-mentioned subclusters within the SN cluster and the psychopathological, cognitive, and functioning variables are shown in Table 2. The rsFC values in the SN of both the R-AI and R-VTA showed a negative correlation with the avolition factor of the BNSS and a positive correlation with the PSP scores (Table 2).

## 4. Discussion

To the best of our knowledge, this is the first fMRI study designed to compare the role of VEN-enriched cerebral areas in shaping the rsFC patterns of SZ patients with HCs in vivo. In the whole sample (subjects with SZ and HCs), we identified a frontoparietal functional connectivity network shared by four functional clusters. This result is in agreement with the results obtained by Cauda et al. [29] using a homologous methodology in a sample of twenty healthy subjects. In addition, we found a significant difference between the rsFC patterns derived from VEN-enriched areas of patients with SZ and HCs. This difference emerged exclusively within a functional cluster superimposable with the SN. This functional network, which involves the insula, the ACC, the supplementary motor area, and the precentral and fusiform gyri [57], is considered a transitional network that links cognition and emotion or interoception [58]. Its activity is thought to regulate cognitive resource allocation by perceiving emotionally relevant stimuli [59] and its alteration has been found in different mental disorders, especially SZ [60]. In this disorder, a reduction in the gray matter volume of the area belonging to the SN [59,61] and an altered rsFC within the network itself, as well as between the SN and other functional networks, principally the default mode network (DMN) and the central executive network (CEN) [59,60,62,63], have been observed. In particular, alterations in the resting-state time-varying engagement of the SN with the CEN and the DMN have been proposed as a diagnostic biomarker for schizophrenia [64]. Other brain areas, i.e., the substantia nigra, the VTA, and the striatum, appear to regulate salience in terms of the responses to unexpected cognitive and motivational, aversive, and rewarding stimuli [60]. These subcortical areas are part of the dopaminergic system and their abnormally elevated activity in terms of dopamine levels or rsFC has been associated with a heightened attribution of salience to ordinary experiences and, consequently, positive symptoms (i.e., delusion and hallucinations) of SZ [60,65]. Simultaneously, other studies demonstrated that a hypo-rsFC of these subcortical areas has been associated with the avolition dimension of negative symptoms, probably through the mediation of a deficit in motivational salience, i.e., the cognitive process that propels behavior toward or away from a particular stimulus [55,66,67,68,69].

In our sample of patients with SZ, the right AI, VTA and vSr, and bilateral dACC/pre-SMA appeared less connected within the SN. The R-FIC and R-VTA were significantly correlated with the avolition dimension of negative symptoms and the personal and social functioning of patients. In particular, two significant correlations were found with the avolition factor of the BNSS, including experiential negative symptoms (i.e., avolition, anhedonia, and asociality) and the rsFC within the SN of the R-FIC (r = −0.869; *p* < 0.01) and R-VTA (r = −0.778; *p* < 0.01). These negative correlations indicate a strong association between the reduction in the functional connectivity of these two cerebral areas within the SN and the severity of these disabling negative symptoms. These results are consistent with previous rs-fMRI studies on SZ that analyzed the rsFC by choosing different ROIs as seeds [55,70,71]. Therefore, our findings suggest that a dysfunction in cerebral areas with high densities of VENs may be related to the severity of the experiential negative symptoms. Moreover, we found two other significant correlations between the PSP score and the rsFC within the SN of the R-FIC (r = 0.694; *p* < 0.05) and R-VTA (r = 0.731; *p* < 0.05). These positive correlations describe a strong association between higher values of the rsFC of the R-FIC and R-VTA in the SN and better personal and social functioning of patients with SZ. These results are in agreement with those of Tian et al., who demonstrated a positive correlation between a more “typical” (similar to HC) rsFC anterior–posterior gradient of the insula and the Social and Occupational Functioning Assessment Scale (SOFAS) score of the patients [71]. In this case, better functioning of the VEN-enriched cerebral areas in terms of greater connectivity within the SN may have a positive impact on the functioning of people with SZ. These data seem to be consistent with the association found between post-mortem high concentrations of VENs and the condition of super-agers characterized by above-average cognitive functioning in relation to age [28].

The main limitation of this study is the spatial resolution of the fMRI, which did not allow for the direct investigation of the activity of VENs at a microscopic scale, as the ROIs selected contained a mix of glial cells and neurons different from the VENs. However, it should be noted that in agreement with Cauda et al. [29], these cortical areas, including the majority of VENs, demonstrated a specific rsFC, which appeared partially altered in patients with SZ. Another important restraint is the relatively small sample size, which limits the statistical power of the study. Therefore, these results must be understood as preliminary and require confirmation from studies with greater sample sizes. Moreover, the absence of a clinical control group limits the specificity of our results, as resting-state functional abnormalities of VEN-enriched cerebral areas may also be present in other neuropsychiatric conditions such as bipolar disorder (BD), ASD, AD, and bv-FTD. This is consistent with the results of post-mortem studies, where VEN alterations were found in most of these conditions, not just SZ. New studies with a clinical control group, e.g., patients with BD or bv-FTD, are needed to test whether our findings are specific to SZ. Finally, the lack of a task did not allow us to compare the resting-state functional connectivity of VEN-enriched cerebral areas during the resting state with that during a task. Despite its limitations, this study has some important strengths. First of all, as cortical regions with high densities of VENs were used as seeds for our analysis, we believe that the disconnectivity found within the SN may be related to the altered activity of VENs in subjects with SZ. However, further in vivo and post-mortem studies are needed to confirm this relationship. Moreover, the use of updated tools for psychopathological and cognitive assessment, the enrollment of clinically stable patients, and the choice of a proper control group of HCs can enhance the validity of the methods.

In conclusion, this study provides some evidence to show that in vivo, VEN-enriched cortical areas are associated with an altered resting-state brain activity in people with SZ. These alterations seem to mainly concern the SN and correlate with experiential negative symptoms, as well as personal and social functioning in the real world.

## Figures and Tables

**Table 1:** Demographic, clinical, and cognitive characteristics.

	SZ Group	HC Group	Statistic F/χ^2^	*p* Value
**Sociodemographic variables**				
Age, Years	41.5 (11.3)	42.1 (10.8)	0.003	0.954
Gender, M/F	13/7	13/7	0.000	1
Education, Years	13.7 (4.3)	13.85 (4.2)	0.012	0.912
**Clinical variables**				
Duration of Illness, Years	14.60 (9.81)			
PANSS–Positive, Score	7.45 (1.82)			
PANSS–Disorganization, Score	6.25 (2.63)			
BNSS avolition	21.85 (9.22)			
BNSS expressive deficit	11.65 (7.40)			
CDSS, Total score	4.10 (5.08)			
PSP, Score	61.60 (13.05)			
CPZ Equivalent, mg/d	371.80 (144.87)			
Concomitant SSRI and SM, N {%}	7 {35}			
Concomitant BDZ and NBDZ, N {%}	5 {25}			
SAS, Total score	0.75 (2.15)			
**Cognitive Performance**				
BACS, Verbal memory	38.56 (9.94)	47.11 (7.78)	9.171	0.004
BACS, Verbal fluency	33.05 (11.74)	46.81 (11.10)	14.499	<0.001
BACS, Digit sequencing	15.49 (4.59)	20.96 (4.17)	15.564	<0.001
BACS, Symbol coding	39.01 (11.12)	54.88 (10.16)	22.216	<0.001
BACS, Token motor	66.96 (17.28)	91.55 (8.09)	33.226	<0.001
BACS, Tower of London	17.26 (5.09)	19.50 (3.98)	2.402	0.129
SST, Correct answers	3.85 (1.90)	5.45 (0.69)	12.552	0.001

SZ: patients with schizophrenia; HC: healthy controls; (SD): standard deviation; M: males; F: females; PANSS: Positive and Negative Syndrome Scale; BNSS: Brief Negative Symptoms; CDSS: Calgary Depression Scale for Schizophrenia; PSP: Personal and Social Performance Scale; CPZ: chlorpromazine; SSRI: selective serotonin reuptake inhibitor; *n*: number of subjects; SM: serotonin modulator; BDZ: benzodiazepine; NBDZ: nonbenzodiazepine; SAS: Simpson–Angus Scale; BACS: Brief Assessment of Cognition in Schizophrenia; SST: Strange Stories Test.

**Table 2:** Correlations between the rsFC of SN area and clinical and cognitive characteristics of the SZ subjects.

Clinical/Cognitive Variables	R-vSr	R/L-dACC/ pre-SMA	R-VTA	R-FIC
PANSS–Positive, Score	−0.088	−0.028	−0.086	0.138
PANSS–Disorganization, Score	0.485	0.484	0.228	0.420
BNSS avolition	−0.353	−0.649	−0.869 **	−0.778 **
BNSS poor emotional expression	−0.213	−0.344	−0.379	−0.523
CDSS, Total score	−0.021	−0.285	−0.457	−0.380
PSP, Score	0.189	0.499	0.731 *	0.694 *
BACS, Verbal memory	−0.080	0.317	0.274	0.458
BACS, Verbal fluency	0.149	0.118	0.165	0.356
BACS, Digit sequencing	−0.220	0.139	0.121	0.066
BACS, Symbol coding	0.092	0.324	0.402	0.334
BACS, Token motor	−0.046	−0.146	0.015	−0.013
SST, correct answers	−0.516	−0.381	−0.266	−0.470

* *p* < 0.05; ** *p* < 0.01 after Bonferroni’s correction. PANSS: Positive and Negative Syndrome Scale; BNSS: Brief Negative Symptoms; CDSS: Calgary Depression Scale for Schizophrenia; PSP: Personal and Social Performance Scale; BACS: Brief Assessment of Cognition in Schizophrenia; SST: Strange Stories Test.

## Data Availability

Due to the anonymity guaranteed in the informed consent paperwork at the time when data were collected, data cannot be publicly shared and are controlled by the Comitato Etico Interaziendale of the A.O.U. Città della Salute e della Scienza di Torino. Researchers who wish to request access to these data may contact the corresponding author (claudio.brasso@unito.it).
